# Olanzapine induced tardive dystonia

**DOI:** 10.4103/0253-7613.44158

**Published:** 2008-10

**Authors:** Ashish Aggarwal, R. C. Jiloha

**Affiliations:** Department of Psychiatry, G. B. Pant Hospital and Maulana Azad Medical College, New Delhi, India

**Keywords:** Olanzapine, tardive dystonia, atypicalantipsychotics

## Abstract

Advent of atypical antipsychotics was thought to be a major advancement in the psychopharmacology for schizophrenia. It was thought that these drugs would have low propensity to induce extrapyramidal symptoms including tardive movements. Olanzapine is a thienobenzodiazepine derivative, second generation (atypical) antipsychotic agent. Compared to typical antipsychotics, it has a greater affinity for serotonin 5-HT2A than dopamine D2 receptors, with preferential action at mesolimbic than nigrostriatal dopaminergic pathways. However, only few reports of olanzapine induced tardive dystonia (TD) are available in the literature. We wish to report another case of TD, in a male patient with schizophrenia, which developed after 15 months of treatment with olanzapine.

## Introduction

Extra-pyramidal symptoms (EPS) and tardive syndromes are commonly associated with the use of typical antipsychotic drugs. Tardive dystonia (TD), a very rare side effect induced by antipsychotics, is characterized by local or general sustained, involuntary twisting movements, generally slow, which may affect the limbs, trunk, neck, or face.[1] Tardive dystonia is usually disabling and persistent, and treatment seldom results in satisfactory relief or remission of symptoms.[2] As compared to tardive dyskinesia, TD develops at a younger age and after shorter exposure to antipsychotic drugs. The estimated prevalence of TD with typical antipsychotics is 3% in a clinical population.[3]

Olanzapine, a thienobenzodiazepine derivative, is a second generation (atypical) antipsychotic agent, with a low propensity to inducing tardive dystonia (TD). Compared with typical antipsychotic drugs, it has a greater affinity for serotonin 5-HT_2A_ than dopamine D_2_ receptors. Olanzapine is thought to have preferential action at mesolimbic over nigrostriatal dopaminergic pathways and is, therefore, associated with a very low incidence of EPS than observed with typical antipsychotic drugs.[4] Furthermore, a retrospective analysis of controlled multicentric trials and numerous case reports of patients with psychotic disorders, including schizophrenia, suggested that olanzapine also improves preexisting symptoms of tardive movements.[4] Till today, very few reports of Olanzapine-related TD are available in the existing literature.[5–8] We wish to report a case of TD, which developed after 15 months of treatment with olanzapine.

## Case Reports

A 23-year-old unmarried male, educated up to the sixth standard was suffering from schizophrenia (Diagnostic and Statistical Manual- IV Criteria) for the last three years. His illness began with symptoms of violent abusive behavior, suspiciousness, muttering to self continuously, with disturbed biological functioning and socio occupational decline. Initially (two months after the onset of illness), he was treated with Risperidone up to 6 mg per day, along with lorazepam 4 mg per day. While on Risperidone, the patient developed akathisia, which resolved with 40 mg of propanolol. Also, the patient developed extra-pyramidal symptoms like tremors and rigidity, which required trihexyphenidyl up to 4 mg per day. With this treatment, the patient improved considerably in about three month's time. The patient then started going to work and was doing well, though on occasions he would be seen to be withdrawn and would generally not interact with others. The patient stopped treatment on his own, after about one year of the start of the illness. The patient apparently continued being well for another six months. After this, the patient again had an exacerbation of the illness, with similar complaints along with grandiose delusions. Mental status examination at that time revealed a young aged male with restricted affect, delusions of persecution, grandiose delusions and second person hallucinations. His past, personal and family history was not significant. In view of, a past history of EPS and akathisia with risperidone, Olanzapine was started in the dose of 5 mg, along with lorazepam 4 mg per day. Trihexyphenidyl was also started prophylactically, in a dose of 2 mg per day. Olanzapine was gradually increased to a dose of 20 mg per day. The patient showed marked improvement in the psychotic symptoms after about three months of treatment with Olanzapine 20 mg. Lorazepam was gradually tapered and was given on an ‘as and when’ required basis, for decreased sleep or anger outbursts. Trihexyphenidyl was also stopped within three months of the start of the treatment. The patient was doing well for another year, during which he was on regular follow up and was compliant with the treatment. After about one year and three months (15 months) of olanzapine therapy, episodic abnormal neck movements progres-sively appeared, which, gradually, over the period of the next three weeks, got aggravated with cervical dystonia with retrocollis.

The move-ments were disabling, a source of intense pain, and the patient had to keep his hands behind his head for the support. The movement would decrease when the patient was lying down and absent during sleep.

On observation, the patient's head was turned backwards and on occasions, the patient's head was turning intermittently to the right. Detailed neurological examination, including fundus examination, did not re-veal any other abnormality, including extra-pyra-midal signs or signs of other movement disorders. Trick maneuvers resulted in minimal relief only. Physical examination was within nor-mal limits. The results of routine investigation of blood, serum copper and caerulo-plasmin levels, X-ray of a cervical spine, MRI (magnetic resonance imaging) of the brain and EEG (electroencephalogram) were within normal limits; so were the results of an ophthalmologic examination, including fundus examination.

A diagnosis of antipsychotic induced tardive dystonia was made after excluding other possible causes of dystonia. Trihexyphenidyl up to 8 mg per day was given without any benefit; along with it, the dose of Olanzapine was lowered. Finally, both Olanzapine and trihexyphenidyl were tapered and clozapine was started at a dose of 25 mg/day, with blood monitoring. It was gradually increased to 125 mg/day, over the next one month. This was maintained for another four months, during which the patient gradually started showing improvement in cervical dystonia, without any relapse of psychotic symptoms. Currently, the patient is showing sustained improvement on the same dose for the last one year.

## Discussion

Burke *et al.*[1] defined TD as:

1. Presence of chronic dystonia
2. History of antipsychotic drug treatment
3. Exclusion of known causes of secondary dystonia
4. Negative family history for dystonia

In our observation, the medication period of 15 months and the relatively long time between development of TD and withdrawal of the risperidone, led to our decision that the TD was secondary to Olanzapine.

Our report suggests that past history of antipsychotic-induced EPS may be an important risk factor for developing TD with atypical antipsychotic drugs, although evidence is inconsistent even with typical antipsychotic drugs. This can be inferred from previous case reports also, where patients developing TD had earlier history of drug induced EPS.[6,7]

One of the previous case reports[5] reported TD in a female patient, who was a case of bipolar disorder and also had tardive dyskinesia.

Regarding the pathophysiology of TD, Olanzapine has a D2 receptor occupancy higher than that of clozapine or quetiapine and similar to that of risperidone, which may have accounted for the development of tardive dystonia,[9] though the exact pathophysiological mechanisms are still not clear.

In our case, TD did not respond to anticholinergic medication and there was substantial improvement with clozapine. Available literature supports the use of clozapine in the management of TD,[10,11] although TD has been reported to occur with clozapine also.[12,13] Other reports in the literature suggest improvement with Olanzapine,[14,15] risperidone[16] and quetiapine.[8] This further suggests that the etiology of TD is multifaceted. Also, their might be individual susceptibility for developing antipsychotic induced side effects including TD.

In conclusion, our report illustrates the possibility of developing tardive dystonia with Olanzapine. Therefore, until further data are available, careful assessments are required for movement disorders in patients receiving atypical antipsychotics. Also, more data is required to know the typical characteristics and risk factors associated with Olanzapine-induced TD.
